# Collectanea

**Published:** 1831-10

**Authors:** 


### COLLECTANEA.

**Floriferis ut apes in saltibus omnia libant,**
**Omnia nos, itidem, depascimur aurea dicta.**

### PATHOLOGY.

*Hæmatemesis dependent upon Disease of the Liver.* By Robert Law, A.M. M.B. &c.

Mary Freyne, aged forty-three, married, four days since was suddenly seized with vomiting of blood, and had bloody discharges from the bowels, which continued up to the period of her admission into hospital. On the day on which she was admitted, she vomited not less than a quart of coagulated blood, and exhibited all the symptoms characteristic of such a loss; countenance pale and exsanguinous; lips livid; expression anxious; temperature of lower extremities below the natural state; surface of the body bedewed with cold clammy perspiration; pulse frequent and feeble; fluttering of the heart; voice faultering, (*entrecoupée.*) (℞. Infusi Rosæ ℥v.; Sulphat. Magnes. ℥ss.; Acid Sulphuric dilut. ʒss.; Tinct. Digitalis, gtts. xxx. Misce. sumat unciam 3tiis. horis, vini rubri ℥vi. Legs to be wrapped in flannel; jars of hot water to be applied to the feet.)

November 28. Vomited very little blood, but had frequent tarry discharges from the bowels; seemed quite exhausted; pulse very small and thready; surface of the body cold; countenance anxious; frequent sighing; all her symptoms bespoke approaching dissolution. Wine not seeming to revive her, I substituted French brandy: she died in the course of the evening.

Examination fifteen hours after death: Body not in the least emaciated; lungs quite healthy; heart soft, flabby, and pale, containing a small quantity of fluid blood; a small quantity of serous fluid in the abdomen; the stomach contained about a pint of blood, and the intestines much of the black tarry matter which was discharged by the bowels. The entire tract of the gastro-intestinal mucous membrane, so far from exhibiting any unusual vascularity, seemed quite blanched.

The liver presented an irregular tuberculated or granulated surface; was contracted in size; its anterior margin much less acute than natural. A section of it exhibited small round bodies of various dimensions, separated by dense fibro-cellular septa: this fibro-cellular tissue seemed to be the proper cellular tissue of the organ increased in density, furnishing loculi or capsules to these roundish bodies, which are probably the acini in a state of hypertrophy; these bodies adhered loosely to their capsules, and could be easily detached from them; the consistence of the organ was greater than natural; its colour a whitish grey.

Case II. Peter Quinn, aged thirty-eight, waiter at an hotel, was admitted into hospital November 1827, for vomiting of blood, which was soon followed by ascites; the ascites yielded to mercurial medicines and vegetable diuretics: he was discharged cured in January, and continued well till the ensuing September, when he was again seized with vomiting of blood, and with dropsical swellings, not only of the abdomen, but also of the lower extremities and the scrotum; his breathing was short and laboured; the entire surface of the body was of a yellow jaundiced hue.

Every species of medicine likely to relieve his symptoms was employed, but failed to make the slightest impression on the disease; and the distress of respiration became so urgent, that I directed the operation of paracentesis abdominis. Peritoneal inflammation set in twelve hours after the operation, which I attribute more to his being allowed to remain all night in sheets wet with the discharge from the puncture, than to the operation itself. He only survived the attack thirty-six hours.

Examination twenty-four hours after death: Body not emaciated; lungs healthy; heart small and flabby.

A large accumulation of straw-coloured serum in the cavity of the peritoneum; peritoneum investing the anterior parietes of the abdomen and the intestines highly injected and thickened.

Mucous membrane of the stomach exhibited a general red injected appearance; that of the intestinal canal rather pale.

The liver presented the same irregularity of surface and deviation from natural structure as I have described in the preceding case. I could adduce two other instances whose symptoms during life, and appearances after death, exactly resembled those I have already detailed: death was caused by hemorrhage in each, and examination exhibited the granular condition of the liver.

These cases unequivocally prove the vomiting of blood to depend upon the peculiar alteration of structure of the liver, which was found to exist in all; and it is remarkable, that, although almost every pathologist has noticed this deviation from natural structure in the liver, Frank seems to be the only one who has observed how often it and hæmatemesis stand to each other in the relation of cause and effect.

This diseased condition of the liver would seem to consist in an entire change of the proper substance of the organ, into a dense fibro-cellular tissue, forming a kind of pulp or parenchyma, in which the roundish bodies (which projecting on the surface, give the organ its irregular appearance,) are imbedded; its specific weight is greater than natural, while its actual size is less, and so far from passing the margin of the ribs, it seems to have receded within its ordinary bounds, shewing how fallible a criterion of the health of the organ is either its size or its descent below the ribs; its form is altered; it becomes more round; its anterior margin more obtuse; its division into lobes less defined; its peritoneal covering thicker and more opaque than natural; its section exhibits no trace of blood-vessels, which we may presume to be either compressed or obliterated by the altered structure; hence the impediment to the vena portæ pouring its blood into its ordinary channels, and the influence of the obstruction reverts upon all the branches which concur to form this vein, and thus we do satisfactorily account for the hemorrhage: this fact would tend to confirm an opinion of the ancients respecting the office of the liver; that it was a diverticulum or reservoir of the abdominal venous circulation. As this pathological condition of the liver is most frequently met with in persons who have indulged freely in the use of ardent spirits, and as we know that the direct tendency of such an indulgence is to produce a chronic inflammation of the gastro-intestinal mucous membrane, would it be pushing conjecture too far to suppose the disease of the liver to depend upon the extension of inflammation, from the intestine along the ductus communis choledochus?

The period of life most subject to this disease is from thirty to forty-five; young females, whose menstruation is irregular, suffer profuse discharges of blood from the stomach with impunity; but in these instances, judging from the situation of the pain which precedes the discharge, and from the description which they gave of the sensation they experience, as it were, of a sponge distended with moisture pressing out the lower ribs on the left side, it would seem as if the spleen were relieving itself from a temporary congestion. Here, too, neither the dropsy nor jaundiced hue of the skin are present.

It unfortunately too often happens that the disease of the liver which we have been considering is so insidious in its progress, and excites the alarm of the constitution so little, that either dropsy or hæmatemesis gives us the first intimation of its existence, when unhappily the organ has undergone such alteration of structure, as to render the effects of medicine at least uncertain: should vomiting of blood first bring it under notice, this alarming complication will be the absorbing object of our attention; and though our patient survive this first attack, we cannot calculate on its not soon returning again, when we consider the permanency of the cause on which it depends. We next have to grapple with the original disease, and its consequent dropsy, and not unfrequently with a chronic inflammation of the peritoneum, which often complicates this morbid series. The treatment which I employed in Quinn's case, and which was followed by at least a temporary success, consisted of blue pill combined with Dover's powder, and frictions on the right side with an ointment composed of mercurial ointment and hydriodate of potash, vegetable diuretics and occasional warm baths: these means, steadily pursued for six weeks, accomplished the cure of the dropsy; and this treatment, too, I should generally adopt in similar cases, as most calculated to meet the several indications of this morbid complication.

Since the preceding paper has been submitted to the notice of the Association, two other instances of this granulated condition of the liver have come under my observation; both laboured under ascites and general dropsy; one died of hæmatemesis, and the other sunk after tapping the abdomen. The subject of this latter case, in detailing the history of his illness, stated that he had had repeated vomitings of blood since its commencement: thus, in six instances, have I observed the coincidence of hæmatemesis and granular liver, from whence I conceive we are warranted in considering, that the discharge of blood depended upon the altered structure of the liver. I have further observed, that this is the only diseased condition of the liver which gives rise to vomiting of blood.—*Dublin Med. Transactions.*

---

### MISCELLANEOUS.

### WESTMINSTER HOSPITAL.

*Femoral Aneurism opened by Mistake. (Abstract of a Clinical Lecture delivered by* Mr. Guthrie, *September 17th.*)

The case to which I am desirous of drawing your attention has terminated fatally, as I expected. It was that of a young man, Thomas Cabley, twenty-seven years of age, by trade a bookbinder: it appears that he had some sort of swelling at the upper part of the thigh, seven years back, for which he applied to a practitioner for advice; but as it gave him little trouble, he did not attend to it until within the last three months, when it enlarged, became painful and pulsating, and the limb proportionally weak. The surgeon he applied to poulticed it for three weeks, and, on Monday the 5th, he opened it with a lancet; but, as only blood followed, the opening was closed by compress and bandage. On Thursday the 8th, it burst out bleeding, and the man says he lost four pints of blood: this is probably an exaggeration, but, from his pale and depressed appearance, it is likely that he lost a considerable quantity. On Saturday evening he was sent to the hospital, and on Sunday morning I proceeded to place a ligature on the external iliac artery. Mortification of the extremity followed, and he died on Tuesday night, at eight o'clock.

This is the second case I have had of femoral aneurism sent into the hospital, after it had been opened. I do not believe that in either case it occurred through ignorance, but from inattention; from the practitioner having made up his mind that they were abscesses, and then proceeding in their treatment as such without further consideration.

These cases will shew you the necessity that exists for a patient investigation of disease, wherever it may take place, and more particularly when situated among parts of importance. It would have been impossible to mistake this last case, if the slightest examination had been made to ascertain whether it was or was not an aneurism; for the pulsation was distinct, and the peculiar whizzing noise was clearly perceptible to the ear. In the first case, the aneurism was in the situation of the artery just as it passes through the tendon of the triceps; and, as the man was old, I preferred doing one operation only, and amputate the limb, but he did not recover. In the present case, the tumor extended up to and beneath Poupart's ligament, near the ilium: it was as large as an infant's head, and was found to contain, after death, eighteen ounces of coagulated and grumous blood, besides about eight ounces of adherent coagula. The artery had given way exactly opposite to the origin of the profunda. Amputation could only have been done at the hip joint, and I preferred the operation of tying the external iliac artery, which I found a difficult undertaking; so much so that one of the students, who has seen me do the operation several times on the dead body at lecture, expressed his great surprise at the different appearance the parts assumed. The limb could not be bent in the slightest degree, without giving the man intolerable pain, and the tumor pushed up Poupart's ligament like a bank, so that the artery, after the external incisions were made, was at a considerable depth, and perfectly tense. Bending the body only brought down the peritoneum, with its contents, so as to completely obscure the aneurismal needle, which was, however, passed under the artery, but with much greater difficulty than is usually experienced. This difficulty occurred from the fascia covering it being very strong, and from the vessel being upon the stretch. It was secured with a single ligature, when the pulsation in the tumor ceased. The temperature of the limb before the operation was 97½° in the ham, and in the opposite, or right ham, 101°. The operation was done at ten in the morning: at three o'clock P.M. the temperature of the left foot was 86½°; at seven in the evening, it was 96°; that of the other foot, 102°. The patient complained of pain in the thigh and in the calf of the leg and heel, which I considered very unfavorable, and expressed my fear that mortification was about to take place. The pulse was 120; the temperature was maintained by the aid of bottles filled with hot water, and by flannel, and an opiate was administered. The next morning the mottled appearance of the leg was decisive of gangrene having supervened; and on the subsequent evening he died, the gangrene having extended up to the tumor. The external iliac, where the ligature was applied, was sound; the aorta was unaffected.

I have elsewhere given it as my opinion, that the operation on an old aneurism is more likely to be successful than on a recent one, of two or three weeks' formation; but a small aneurism is always more favorable for the operation than a large one; and here it was too large: it pressed on the collateral branches; it pushed aside and pressed on the great vein, diminishing, I believe, in consequence, the temperature of the limb before the operation, and being the principal cause of the mortification which ensued. If the circulation through the limb had been preserved, the patient had still many dangers to encounter, all depending on the opening which had been made into the sac. The first would arise from the reflux blood finding its way into the sac, and flowing through the wound: now this would not be, in all probability, of an arterial colour, it would be black; a fact of great importance, and which should never be forgotten, as it bears, in an especial manner, on wounded arteries, in which it is always so. I have seen the artery tied higher up when such an occurrence took place, but it did not, of course, succeed; and subsequently the lower end of the divided vessel, which was discharging black blood, had to be sought for and secured.

In aneurism this is not always the case, but it is frequently so: we had an instance to the contrary in this hospital some time ago, and I will take an opportunity of shewing you the preparation. A man came in with an aneurism in the leg, which had burst and distended the limb in every direction. It was thought advisable by his surgeon to make an incision into the swelling, to ascertain its nature: coagulated blood rolled out, and presently arterial blood in a full jet. This I could prevent by compression; but not a steady flow of it, without any impetus, which came from the lower end of the artery, and amputation was resorted to, but it was not successful. In this case the collateral branches were large, and the communication nearly direct, the artery having burst in the upper part of the calf. If the coagulation in the blood in the sac should have prevented this accident, still, from the state of the parts, suppuration must have taken place: it had, indeed, actually commenced on the outside of the fascia forming the sac, and the whole contents would have been gradually discharged. An operation in the lower extremity of the artery would have become necessary, if the inflammation had not extended to it, so as to fill it with coagulum; and, lastly, the drain from this great hole would, in all probability, have destroyed the patient, in his reduced state. All these evils would have been caused by the error of making a small opening into the sac, and shews you the importance of a careful diagnosis.

A patient does not always, however, die under these circumstances. A man was brought into this hospital some five years ago, with a large, deep, sloughing hole on the anterior and upper part of the thigh, precisely in the situation of this aneurism. From the history of the case, I had no doubt of its having been an aneurism, which had sloughed, and was undergoing a spontaneous cure: it was not, however, effected, for the capsular ligament of the hip-joint ulcerated, and he died from disease of the joint. On dissection, the artery was found to be deficient for several inches.

Mr. Lynn has mentioned to me, that, many years ago, a man was brought into the hospital, with an aneurism in each thigh, which burst at once, and so much blood was lost that he was left in an apparently dying state. The hemorrhage did not return; the man gradually recovered, and a spontaneous cure of the aneurism followed.

Independent of the history of the case, and many collateral circumstances, there are two great distinguishing marks of an aneurism, the pulsation and the sound. An aneurism always gives a feeling of pulsation, unless it be filled by coagulum and be impervious to the passage of blood through it, which are rare occurrences; or when it bursts, and a quantity of blood is extravasated between the muscles, in the cellular structure of the limb, and under the facia. In this latter case, the sound of the blood passing through the ruptured vessel may be heard, and it is peculiar.

I drew your attention several times last spring to a remarkable case among yourselves. One student thought it right to bleed another, and taking it for granted that an elastic vessel, which he felt under the skin after he had applied a bandage, was a vein, he opened it, and, on loosening the bandage, abstracted the desired quantity of arterial blood: this alarmed him, and, on becoming aware of his error, he placed a solid compress on the part, and brought his friend two days after to me. It was the ulnar artery, running superficially, that he had opened: the external wound was closed, and a small aneurism, the size of the tip of the little finger, had formed. On applying the ear to this, the sound emitted was similar, and just as great as that which would fall on the ear from a bellows in a smith's forge; and I judged of the progress towards cure through the obliteration of the artery at that part, by the gradual diminution of the sound. The application of the ear to a swelling in the situation of an aneurism is indispensable; and it will be found, I do not hesitate to say, decisive in most instances of even a very doubtful nature.

Mr. Guthrie then called in a woman labouring under aneurism of the arch of the aorta, and directed the attention of the students to the extent of pulsation under both clavicles, and the single sound, like the stroke of a hammer, which it communicated to the ear, when compared to the double sound of the heart. This woman, he said, came to the hospital about five years ago, with a small aneurism presenting at the right sterno-clavicular articulation: it was called a subclavian aneurism, and the operations on the carotid and subclavian were recommended for its cure. Mr. Guthrie believed he had been blamed for not operating, but he was even then satisfied that the disease was of the aorta. This woman has since had a child, and may yet live to have more; and, upon the whole, Mr. Guthrie believed forbearance to have been the safest and most judicious course of proceeding.

